# Correction: The Brazilian vaccine divide: How some municipalities were left behind in the Covid-19 vaccine coverage

**DOI:** 10.1371/journal.pgph.0003447

**Published:** 2024-06-25

**Authors:** Antonio Fernando Boing, Alexandra Crispim Boing, Lorena Barberia, Marcelo Eduardo Borges, S. V. Subramanian

The fifth author’s name is spelled incorrectly. The correct name is: SV Subramanian.
